# Genome-wide linkage analysis of age at onset of alcohol dependence: a comparison between microsatellites and single-nucleotide polymorphisms

**DOI:** 10.1186/1471-2156-6-S1-S12

**Published:** 2005-12-30

**Authors:** Bamidele O Tayo, Yulan Liang, Saverio Stranges, Maurizio Trevisan

**Affiliations:** 1 Department of Social and Preventive Medicine, University at Buffalo, Buffalo, New York, USA; 2 Department of Biostatistics, University at Buffalo, Buffalo, New York, USA

## Abstract

**Background:**

Using the dataset provided for Genetic Analysis Workshop 14 by the Collaborative Study on the Genetics of Alcoholism, we performed genome-wide linkage analysis of age at onset of alcoholism to compare the utility of microsatellites and single-nucleotide polymorphisms (SNPs) in genetic linkage study.

**Methods:**

A multipoint nonparametric variance component linkage analysis method was applied to the survival distribution function obtained from semiparametric proportional hazards model of the age at onset phenotype of alcoholism. Three separate linkage analyses were carried out using 315 microsatellites, 2,467 and 9,467 SNPs, spanning the 22 autosomal chromosomes.

**Results:**

Heritability of age at onset was estimated to be approximately 12% (*p *< 0.001). We observed weak correlation, both in trend and strength, of genome-wide linkage signals between microsatellites and SNPs. Results from SNPs revealed more and stronger linkage signals across the genome compared with those from microsatellites. The only suggestive evidence of linkage from microsatellites was on chromosome 1 (LOD of 1.43). Differences in map densities between the two sets of SNPs used in this study did not appear to confer an advantage in terms of strength of linkage signals.

**Conclusion:**

Our study provided support for better performance of dense SNP maps compared with the sparse mirosatellite maps currently available for linkage analysis of quantitative traits. This better performance could be attributable to precise definition and high map resolutions achievable with dense SNP maps, thus resulting in increased power to detect possible loci affecting given trait or disease.

## Background

Several reports have been published on the use of evenly spaced microsatellites, usually less than 500, for genome-wide linkage analysis of traits or diseases with reasonable heritability. Although this has lead to great successes in mapping of Mendelian disorders, comparable successes still remain to be achieved in complex disease mapping [1]. It is no wonder, therefore, that other possible approaches and tools are constantly being employed in the search for genes that may influence complex traits or diseases. One of these tools is single-nucleotide polymorphisms (SNPs) which, although biallelic, when mapped in a highly dense manner are able to provide at least approximately the same amount of information as the common set of microsatellites [2]. The abundance of this type of genetic marker in the genome and the availability of highly automated genotyping procedures have made it increasingly possible to obtain highly dense SNP maps with very high information content compared with the current standard densities used in microsatellite maps for linkage analysis. To explore the utility of dense SNP maps compared with the limited number of microsatellites often available for linkage analysis, we carried out genome-wide linkage analysis of age at onset of alcoholism in the Collaborative Study on the Genetics of Alcoholism (COGA) data provided for Genetic Analysis Workshop 14 (GAW14) using 315 evenly spaced microsatellites, 2,467 Illumina SNPs, and 9,467 Affymetrix SNPs all separately spanning chromosomes 1 to 22.

## Methods

### Study population

The COGA dataset provided for GAW14 contained 1,614 family members in 143 families with different sizes. Each family was ascertained from an alcoholic proband in a treatment program. Self-reported ethnicity in this study population included American Indian, Asian, Pacific Islander, Black Hispanic and non-Hispanic, and White Hispanic and non-Hispanic. From the list of alcohol-related phenotypes available in the dataset, we chose the age at onset for alcohol dependence (ALDX1). ALDX1 was defined as lifetime diagnosis [3] of alcohol dependence by DSM-III-R (Diagnostic and Statistical Manual of American Psychiatric Association-Revised) [4] criteria and definitive alcoholism by Feighner [5] criteria. Only individuals meeting both criteria were considered affected in this study.

### Modelling age at onset data

Because age at onset is a truncated quantitative trait – censored for those individuals for whom age at onset was not observed, we used semiparametric proportional hazards model as implemented in SAS [6] software to estimate the survival distribution function after adjusting for significant covariates – age, sex, maximum number of drinks in 24-hour period, and smoking status. Our assumption for using this truncated quantitative trait model was that all individuals would eventually get the disease if they lived or if the follow-up was long enough.

### Statistical genetic analyses

There were 315 microsatellites (11.53 cM average spacing), 4,596 Illumina SNPs (1.41 cM average spacing), and 10,801 Affymetrix SNPs (0.36 cM average spacing) provided across the autosomal chromosomes. In cases were there were multiple SNPs per locus, we carried out thinning by random sampling of one SNP per locus and then discarded others. This process thus resulted in 2,467 and 9,467 effective SNP loci for Illumina and Affymetrix, respectively, without affecting the original marker spacing. We then proceeded to use SOLAR [7] software to estimate marker allele frequencies from the data by maximum likelihood estimation method for both the microsatellite and the two sets of SNPs because of detected errors in the original frequency files provided with the data. Marker-specific identity by descent (IBD) for pairs of relatives and multipoint IBD were also estimated using the same software. Multipoint nonparametric linkage analyses across the 22 autosomal chromosomes at 1-cM intervals were performed using variance component procedure as implemented in SOLAR. Separate linkage analysis of age at onset was performed using each set of genetic markers.

## Results

**Figure 1:**
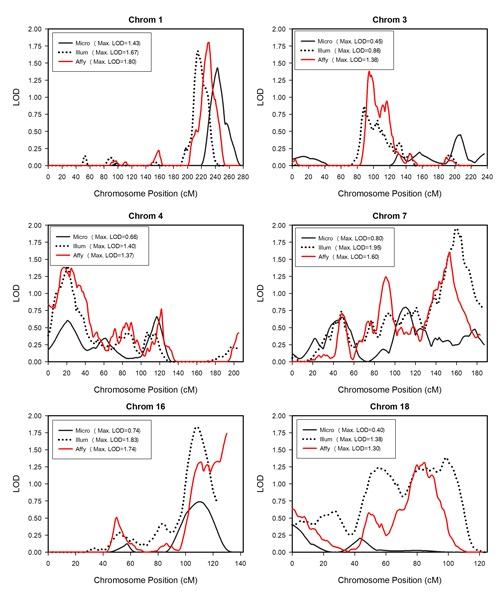
**Linkage signals. **Linkage signals obtained with microsatellite (Micro), Illumina SNP (Illum) and Affymetrix SNP (Affy) maps on selected chromosomes.

**Figure 2:**
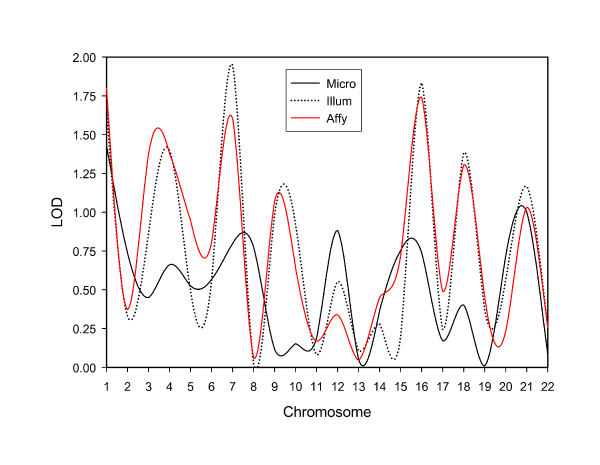
**Plot of highest linkage signals. **Plot of highest linkage signals on 22 chromosomes using microsatellites (Micro, black), Illumina (Illum, dotted) and Affymetrix (Affy, red) maps.

Prior to the multipoint linkage analysis, we fitted a polygenic model and estimated the heritability of age at onset to be 11.8% ± 3.9% (*P *< 0.001). The random environmental effects thus contributed 88.2% of the total variation for this trait. The distributions of the highest multipoint LOD scores (logarithm of the odds to the base 10) and their corresponding chromosome positions for each of the genetic marker sets are presented in Table 1 and Figure 1 for selected chromosomes. The highest LOD score obtained with microsatellites was 1.43 on chromosome 1 at 243 cM (on marker D1S549), with none of the other chromosomes having LOD score greater than 1.00. For the SNPs, the highest LOD scores were 1.95 at 159 cM on chromosome 7 for Illumina, and 1.80 at 230 cM on chromosome 1 for Affymetrix. Other chromosomes with highest LOD scores above 1.00 include 4, 16, 18, and 21 for both Illumina and Affymetrix SNPs; and chromosomes 3 and 9 for Affymetrix SNPs. The distributions of the highest linkage signals on the chromosomes are generally similar for both Illumina and Affymetrix, but different for microsatellites (Figure 2). The difference in chromosome positions for highest linkage signals for the two SNP marker sets on any chromosome ranged from 2 cM to 22 cM (Table 1).

## Discussion

In this study we compared the linkage signals obtained through the use of limited number of microsatellites and two sets of relatively dense SNP maps in nonparametric multipoint linkage of age at onset of alcoholism across the 22 autosomal chromosomes. Although there was neither significant nor suggestive evidence of linkage [8] for age at onset of alcohol dependence from any of the three sets of genetic markers in this study, there were significant differences in the distributions and strength of linkage signals between microsatellites and the SNPs. We observed that the only linkage signal above 1.00 LOD score obtained with microsatellites (LOD = 1.43 at 243 cM) was on chromosome 1, and this was weaker than any of those obtained with Illumina (LOD = 1.67 at 215 cM) and Affymetrix (LOD = 1.80 at 230 cM) SNPs on the same chromosome. Furthermore, linkage signals detected with SNPs on other chromosomes ranged from LOD scores 1.33 to 1.95 at different positions on chromosomes 3, 4, 7, 16, 18, and 21. The highest LOD scores from SNPs are 1.95 on chromosome 7 (Illumina) and 1.80 on chromosome 1 (Affymetrix). There were generally low linkage signal detections by microsatellites compared with SNPs across the genome. One likely reason for this poor comparative performance could be the low information content of the low-resolution microsatellite map. Since high map resolutions, such as those obtained with the two sets of SNPs in this study, also translate into increased information content, it is our belief that the use of highly dense SNP map in linkage analyses would more likely lead to detection of linkage signals that would be missed by the currently available microsatellite map.

As seen in Figure 2, the differences in map densities between Illumina and Affymetrix SNPs used in this study did not appear to confer any advantage in terms of strength of linkage signals. In fact, the Illumina map detected stronger linkage signals than the Affymetrix map on most of the chromosomes in which signals were detected (Table 1). Since it is possible that a less informative marker may be sampled at any given locus with the thinning method used in this study, the observed differences in chromosome positions for highest linkage signals for the two SNP marker sets (Table 1) could have been due to this. However, our results from using the two SNP marker sets without thinning (data not shown) indicated that the observed differences are not due to the thinning method used.

## Conclusion

This study provided support for better performance of dense SNP map compared with microsatellites for linkage analysis, even for traits with low heritability such as age at onset of alcoholism. This better performance is attributable to high map resolution achievable with dense SNP maps. Also, the more abundant genome-wide distribution of SNPs compared with microsatellites, and the availability of advanced genotyping technology to process rapidly and extract needed data from the samples place SNPs as very promising tools for linkage analysis.

## Abbreviations

COGA: Collaborative Study on the Genetics of Alcoholism

GAW: Genetic Analysis Workshop

IBD: Identity by descent

SNP: Single nucleotide polymorphism

## Authors' contributions

BOT conceived of the study, performed all statistical analyses and wrote the manuscript. YL participated in the design, statistical analyses, and preparation of manuscript. SS and MT participated in the design, and preparation of manuscript. All authors read and approved the final manuscript.

## Figures and Tables

**Table 1:** Highest linkage signals and the corresponding chromosome positions in centimorgans on 8 selected chromosomes.

	SNPs		
	
Chromosome	Microsatellite (cM)	Illumina (cM)	Affymetrix (cM)
1	1.43 (243)	1.67 (215)	1.80 (230)
3	0.45 (207)	0.86 (88)	1.38 (95)
4	0.66 (117)	1.40 (21)	1.37 (25)
7	0.80 (110)	1.95 (159)	1.60 (153)
9	0.12 (58)	0.99 (131)	1.07 (125)
16	0.74 (110)	1.83 (108)	1.74 (130)
18	0.40 (1)	1.38 (99)	1.33 (85)
21	1.00 (3)	1.17 (48)	1.03 (50)
